# Supplementary material for: Barriers from Multiple Perspectives Towards Physical Activity, Sedentary Behaviour, Physical Activity and Dietary Habits When Living in Low Socio-Economic Areas in Europe. The Feel4Diabetes Study
Source: Int J Environ Res Public Health. 2018 Dec 13;15(12):2840. doi: 10.3390/ijerph15122840 (PMC6313803; doi:10.3390/ijerph15122840)
Supplement: Supplementary file 1 [file ijerph-15-02840-s001.zip › ijerph-379416-R1 Supplementary_A.pdf]

Semi-structured interview guides for the focus groups conducted within the Feel4Diabetes-study

Interview guide for parents

OPENING QUESTION

Can you please tell us your name and how many children you have?

KEY QUESTIONS FOR <u>GENERAL LIFESTYLE</u>

A healthy lifestyle is important for a good health.

1. Let us begin by asking what you think a healthy lifestyle is?
2. Are you satisfied with your current lifestyle? Why or why not?
3. What would you like to change and do you think this is feasible? Why or why not?
    - If you do not want to change anything, what is the reason for this? What makes you satisfied?
    - What would motivate you or what could be done to change this?

KEY QUESTIONS FOR <u>PHYSICAL ACTIVITY</u>

Being physically active is also important for health. With physical activity we mean activities during which the body is moving, for example during sports (in a sports club or doing sports by yourself, like jogging), or during walking or bicycling. This can be done in your free time, at your work, or as a way to get somewhere, for example when going to your work by bicycle or if you walk to a bus stop.

4. Let us begin by asking whether you spend a lot of time on physical activity? If so, what activities do you do? Why?
5. Are you satisfied with the current amount of physical activity? Why or why not?
6. Do you think there is a difference in time you spend on physical activity on week days and weekend days?

7. Would you like to change something about the amount of time you are physically active and do you think this is feasible? Why or why not?
    - If you do not want to change anything, what is the reason for this? What makes you satisfied?
    - What would motivate you or what could be done to change this?
        - On weekdays?
        - On weekend days?
8. Is your child often physically active? If so, during which activities? For example, is he/she member of a sports club, does he/she bikes a lot, …?
9. Would you like to increase the amount of time your child spends on physical activity? Why or why not?
    - If yes: what is the reason you would like this?
    - If no: why not?
10. Do you sometimes do physical activities together with your child? For example, by going to a park or by swimming together? Why or why not?
11. Can anything be done to increase physical activity together with your child(ren)?

KEY QUESTIONS FOR <u>FACILITIES</u>

To be active, you may need some facilities. Let's start with talking about some facilities in your home environment.

Home environment

12. Which facilities do you have at home to be physically active?
    - If you could change something about this, what would it be?
    - Why would it be difficult to change this?

Neighbourhood

Now, let's talk about the facilities for being physically active in your neighborhood (neighborhood is defined as walking distance of maximum 10-15 minutes).

13. Which facilities do you have in your neighborhood for being physically active? For example, do you have a (public) swimming pool in your neighborhood, or a park, or a playground?

- Would you like to change something about this? Why or why not?

14. Do you frequently use these facilities? Why or why not?
15. Can anything be done to increase your use of these facilities

KEY QUESTIONS FOR <u>SEDENTARY BEHAVIOUR</u>

Now we are going to talk about sitting behaviour. Let us begin by explaining what sitting behaviour is: In general, sitting behaviour is any behaviour during which you are sitting or lying down, like watching TV, reading, using a computer/tablet, sitting at your desk, driving a car, …

16. Do you think you spend a lot of time on sitting behaviour? If so, what activities do you do?
17. Do you think you spend too much time on [watching TV]? Why or why not?
18. Do you think that the time you spend on [watching TV] is different between weekdays and weekend days? Why or why not?
19. Would you like to change something about the amount of time you spend on [watching TV] and do you think this is feasible? Why or why not?
    - If you do not want to change anything, what it the reason for this? What makes you satisfied?
    - What would motivate you or what could be done to change this?
        - On weekdays?
        - Weekend days?

<u>Are there other activities during which you often sit? [Computer/tablet use] should also be questioned. Questions 5, 6, 7 are repeated for [computer/tablet use]).</u>

20. Does your child spend a lot of time on sitting behavior? If yes, during which activities? For example, do they watch a lot TV? Do they play a lot on the PlayStation? Do they often use the computer/tablet?
21. Do you think it is OK that your child spends a lot of time on sitting activities? Why or why not?
    - If yes: Why do you think it is OK?
    - If no: What would you like to change and why?

22. Do you sometimes do sitting activities together with your child? For example, by playing (social) games together? Why or why not?

KEY QUESTIONS FOR <u>EATING BEHAVIOURS</u>

Lastly, we will talk about your family's eating habits.

23. Tell us about your and your family's eating habits. For example, does your family takes breakfast daily? Why or why not?
    - Does your family eat vegetables daily? Why or why not?
        - How many portions do you eat daily, on average?
    - Does your family eat fruit daily? Why or why not?
        - How many pieces do you eat daily, on average?
    - What does your family eat as a snack? Why?
    - What do you usually give along with your children when they go to school? Why?
24. Are you satisfied with your current eating habits? Why or why not?
25. Do you think there is a difference in what you eat on weekdays and on weekend days?
26. What would you like to change and do you think this is feasible? Why or why not? For example, by eating more fruits and vegetables, and less snacks/candies?
    - If you do not want to change anything, what is the reason for this and what makes you satisfied?
    - What would motivate you or what could be done to change this?
27. What do you mainly drink in your family and why?
    - Does your family often drink sugar-sweetened beverages? For example cola, cola light, (pre-packed) fruit juice, chocolate milk
28. What do you prefer that there is drunk in your family and why?
29. Are you satisfied with your and your family's current drinking habits? Why or why not?
30. What would you like to change about your drinking habits, and do you think this is feasible for you? Why or why not? For example no soft drinks during meals.
    - If you do not want to change anything, what is the reason for this and what makes you satisfied?

- What would motivate or what can be done to change this?

KEY QUESTIONS ABOUT THE INFLUENCE OF SCHOOL

31. In a last question, we want to ask whether you think the school has an influence on the lifestyle of your child? Why or why not? For example, does the school has an influence on what your child eats or drinks? With a healthy lifestyle we mean having enough physical activity, having healthy eat- and drinking habits, and a limited time spend on sitting behavior.
    - Do you think it is important that the school influences the lifestyle of your child?
    - Do you think it is the responsibility of the school to teach/learn your child how to live a healthy lifestyle?
        - What would you think when the teacher says to your child to watch less TV at home, to drink more water instead of sugar-sweetened beverages, or eat less unhealthy snacks?

ENDING QUESTION

We are going to create a program that wants to promote healthy eating, healthy lifestyle and physical activity and that wants to decrease sedentary behaviour.

32. Do you think that we, or the city/municipality could change the lifestyle of families? Why or why not?
33. As we design the program, what advice do you have for us?

Interview guide for teachers

OPENING QUESTION

Can you please tell us your name and where you teach?

KEY QUESTIONS FOR PHYSICAL ACTIVITY

Being physically active is also important for health. With physical activity we mean activities during which the body is moving. Think about sports lessons on school, free play. Also walking or cycling as part of transport to school is an example of physical activity.

1. Let us begin with the first question: Do you think your pupils spend a lot of time on physical activities? If yes, during which activities?
2. Are there enough opportunities on the playground for pupils to be physical active during school time? Why, why not?
3. Does your school often organizes sports? For example extracurricular sport activities. Why, Why not?
    - If yes: which initiatives could change this?
4. Are there many pupils who walk or cycle to school? Why or why not?
    - If no: Which initiatives could change this? For example: teachers who guide pupils on dangerous traffic points.

KEY QUESTIONS FOR EATING BEHAVIOURS

Furthermore we would like to talk about eating behaviours of your pupils.

5. Do your pupils bring particular healthy or unhealthy food and/or beverages to school? For example, snacks during break or lunch.
6. Which school rules exist regarding pupil’s eating or drinking behaviour. Why?
7. Can pupils buy (healthy or unhealthy) food and beverages on school? Like vending machines, a school store…
    - Is there a possibility to change the content of the vending machines or the school store?

KEY QUESTIONS FOR SEDENTARY BEHAVIOUR

Now we are going to talk about sitting behaviour. Let us begin by explaining what sitting behaviour is: In general, sitting behaviour is any behaviour during which you are sitting, reading, studying, using a computer/tablet, sitting at your desk

1. Let us begin with the first question: Do you think your pupils spend a lot of time on sitting activities during school time? If yes, when?
2. Do you think it is feasible to change the time they spend on sitting activities? Why or why not?
    - If yes: Which initiatives could change this? For example, less benches on playground, standing desks.
    - If no: Why not?

KEY QUESTIONS FOR <u>INFLUENCE OF SCHOOL</u>

8. To finish, we would like to ask whether you think school has a positive influence on the lifestyle on your pupils, for example on what they eat or drink? Why or why not? With healthy lifestyle we mean healthy eating, being physically active and limit sedentary behaviour
    - Do you think it is the responsibility of a teacher/school to offer a healthy lifestyle to their pupils?

ENDING QUESTION

9. We are going to create a program that wants to promote a healthy lifestyle. Do you think we, or you as teacher, or a city or town can influence the lifestyle of pupils? Why or why not?
10. If we design the program, what advice do you have for us?
    - We should give a number of counseling sessions. Do you have tips for us regarding these counseling session?

Interview guide for local community workers

OPENING QUESTION

Can you please tell us your name and where you work.

KEY QUESTIONS FOR GENERAL LIFESTYLE

A healthy lifestyle is important for a good health. Especially in vulnerable groups, an unhealthy lifestyle seems to be a problem. Vulnerable families are often families with a low-socio-economic status.

1. Let us begin with the first question: Do you think vulnerable families have a healthy lifestyle? Why or why not?
   - What do you think are the most important reasons for families to live a certain lifestyle? Why or why not?
   - Which factors could influence the lifestyle of these families and why? This can be environmental factors, like unsufficient facilities, or personal factors, like unsufficient financial resources, etc.
   - What do you think could motivate these families to live a more healthy lifestyle?
   - Do you think they have enough knowledge about what a healthy or unhealthy lifestyle is? Why or why not?

KEY QUESTIONS FOR PHYSICAL ACTIVITY

Being physically active is also important for a good health. Physical activity is any bodily movement. Think about being active in a club or walking or cycling. You can be physically active in leisure time, at your work or as part of transport. For example cycling to work or walking to a bus stop.

2. Do you think vulnerable families spend a lot of time on physical activities? Why or why not?
3. Do you think it is possible to change the time they spend on these activities?
   - If yes: which initiatives could change it?

- If no: Why not?

KEY QUESTIONS <u>FOR ENVIRONMENT</u>

Let us talk about the opportunities that could stimulate the vulnerable families to be more physically active.

Environment

4. Do you think these families have enough opportunities to be physically active in their neighbourhood? Why or why not?
5. Do you think they often use these opportunities? Why or why not?

KEY QUESTIONS <u>FOR SEDENTARY BEHAVIOR</u>

Now we want to talk about sitting behaviour in vulnerable families. Let us begin with explaining what sitting behaviour is: In general, sitting behaviour is any behaviour during which you are sitting or lying down, watching TV, reading, use a computer/tablet, sitting at your desk, driving a car,…

6. Do you think vulnerable families spend a lot of time on sitting activities? Why or why not?
7. Do you think there it is possible to change the time they spend on these activities?
   - If yes: which initiatives do you think could change it?
   - If no: Why not?

KEY QUESTIONS <u>FOR EATING BEHAVIOURS</u>

Furthermore, we would like to talk about eating behaviours of these families

8. Do you think vulnerable families have particular eating behaviours? Why or Why not?
9. Do you think it is easy to change these eating behaviours? Why or why not?
   - What would motivate vulnerable families to eat more healthy and why do you think so?
10. What are the drinking habits of these families and why?
    - Do they often drink sugar-sweetened beverages like Cola, cola light, (pre-packed) fruit juices chocolate milk?
11. Do you think it is be easy to change these drinking habits? Why or why not?

- What would motivate vulnerable families to drink more healthy and why do you think so?

KEY QUESTIONS FOR INFLUENCE OF LOCAL COMMUNITY CENTERS

12. To finish, we would like to ask you whether you think that the local community centers have an influence on the lifestyle of these families, for example on what they eat or drink? Why or why not? With a healthy lifestyle we mean having healthy eating habits, being enough physically active and limited sedentary behaviour
    - Do you think it is the responsibility of a local community center to offer a healthy lifestyle to these families?
13. We are going to create a program that wants to promote a healthy lifestyle. Do you think we, or you as local community worker, or a city or town can influence the lifestyle of these families? Why or why not?
14. If we design the program, what would you advise us ?
    - We are planning to give a number of counseling sessions.. Do you have tips for us regarding these counseling session?
